# A comparative analysis of linear regression, neural networks and random forest regression for predicting air ozone employing soft sensor models

**DOI:** 10.1038/s41598-023-49899-0

**Published:** 2023-12-16

**Authors:** Zheng Zhou, Cheng Qiu, Yufan Zhang

**Affiliations:** Department of Material and Environmental Engineering, Chengdu Technological University, Chengdu, China

**Keywords:** Environmental chemistry, Scientific data, Statistics

## Abstract

The proposed methodology presents a comprehensive analysis of soft sensor modeling techniques for air ozone prediction. We compare the performance of three different modeling techniques: LR (linear regression), NN (neural networks), and RFR (random forest regression). Additionally, we evaluate the impact of different variable sets on prediction performance. Our findings indicate that neural network models, particularly the RNN (recurrent neural networks), outperform the other modeling techniques in terms of prediction accuracy. The proposed methodology evaluates the impact of different variable sets on prediction performance, finding that variable set E demonstrates exceptional performance and achieves the highest average prediction accuracy among various software sensor models. In comparing variable set E and A, B, C, D, it is observed that the inclusion of an additional input feature, PM_10_, in the latter sets does not improve overall performance, potentially due to multicollinearity between PM_10_ and PM_2.5_ variables. The proposed methodology provides valuable insights into soft sensor modeling for air ozone prediction.Among the 72 sensors, sensor NN_R[Y]C_ outperforms all other evaluated sensors, demonstrating exceptional predictive performance with an impressive R^2^ of 0.8902, low RMSE of 24.91, and remarkable MAE of 19.16. With a prediction accuracy of 81.44%, sensor NN_R[Y]C_ is reliable and suitable for various technological applications.

## Introduction

### Background and importance of air ozone prediction

Air pollution, including compounds such as ozone, has become a global concern due to its detrimental effects on human health and the environment^1,2^. Ozone is a reactive gas formed through complex photochemical reactions involving precursor pollutants such as nitrogen oxides (NO_x_) and volatile organic compounds (VOC_s_)^3–5^. Elevated ozone levels in the atmosphere can contribute to respiratory issues, cardiovascular diseases, and lung inflammation in humans. It can also harm plants, reduce crop yields, and disrupt ecosystems. Accurately predicting ozone concentrations in the air is crucial for effective air quality management and the development of appropriate mitigation strategies. By forecasting ozone levels, policymakers, environmental agencies, and health professionals can take timely measures to reduce exposure and mitigate the potential health and ecological risks associated with high ozone concentrations. This can include implementing emission controls, adjusting industrial activities, and raising awareness among vulnerable populations.

### Soft sensor modeling for air ozone prediction and its significance

Soft sensor modeling, also known as virtual sensing or data-driven modeling, enables the estimation of specific physical or chemical parameters using available data and mathematical models^6–8^. In the context of air ozone prediction, soft sensor modeling involves constructing models using relevant environmental variables such as meteorological data, pollutant concentrations and historical ozone measurements to predict ozone levels in real-time or for future periods. This approach allows for the development of virtual sensors that provide continuous estimates of ozone concentrations, even in cases where physical sensors are not present or practical to deploy^9,10^. The significance of soft sensor modeling lies in its ability to overcome limitations associated with physical sensors, such as cost, maintenance, and limited coverage. Soft sensors offer a cost-effective and flexible alternative for ozone prediction, enabling widespread monitoring and forecasting of ozone concentrations. Furthermore, soft sensor models can be continuously updated and optimized using new data, providing accurate and up-to-date information for decision-makers in air quality management and public health.

### Objectives of the study

The main objectives of this study are to compare and evaluate the performance of different soft sensor modeling techniques for air ozone prediction. Specifically, we will compare the effectiveness of linear regression, neural networks and random forests regression in predicting ozone concentrations. These techniques were chosen due to their widespread usage and demonstrated capabilities in modeling complex relationships in environmental systems. Through this comparative analysis, we aim to identify the most suitable modeling technique for air ozone prediction based on criterion such as predictive accuracy, efficiency and interpretability. Additionally, we seek to explore the strengths and limitations of each modeling approach and provide insights into their practical applications in air quality management and decision-making.

## Literature review

### Overview of linear regression, neural networks and random forests regression

Air ozone prediction has been an important area of research due to the detrimental effects of ozone pollution on human health and the environment^11^. In recent years, several studies have been conducted to develop and evaluate different methods for air ozone prediction. Here, we provide an overview of some key research findings and methodologies.

#### Linear regression

LR (Linear regression) is a popular and widely used modeling technique in statistics and machine learning. It aims to establish a linear relationship between the input variables and the target variable. The model assumes a linear combination of the input features to predict the continuous output variable. The coefficients of these input variables are estimated using various optimization algorithms, such as least squares. LR is simple to implement and interpret, making it a good choice for scenarios with linear relationships between variables. MLR (Multiple linear regression) is a form of LR that is suitable for this case. MLR provides equations linking a number of input variables (x_n_) to a target variable (y) using Eq. (1)^12^.

1 $$ {\text{y}} = {\text{w}}_{0} + {\text{w}}_{{1}} {\text{x}}_{{1}} + \cdots + {\text{w}}_{{\text{n}}} {\text{x}}_{{\text{n}}} $$

where w_0_ is the intercept, w_n_ is a coefficient for x_n_ and n is the number of input variables. Out-of-sample accuracy can be improved by using regularization methods which add a penalty term to the model input variables, shrinking the freedom of the input variable during learning.

Nonlinear extension refers to the use of nonlinear feature functions to transform independent variables in linear regression, in order to capture nonlinear relationships in the data.

In LR, we assume that there is a linear relationship between the independent variables and the dependent variable. However, in real-world data, there may exist nonlinear relationships, where the relationship between the independent variables and the dependent variable cannot be accurately described by a simple linear model.

To address this issue, we can use nonlinear extension. This means applying some nonlinear functions to the independent variables to introduce nonlinear features in the model, in order to better fit the nonlinear relationships in the data.

For example, if there is a quadratic relationship between the independent variable x and the dependent variable y, we can square the independent variable x to obtain x^2^ as a new independent variable, and then use both x and x^2^ as input variables to build a linear regression model. This way, the model can capture the quadratic relationship between x and y.MLR with nonlinear extension(MLR-NE) provides equations linking a number of input variables (xn) to a target variable (y) using Eq. (2).

2 $$ {\text{y}} = {\text{w}}0 + {\text{w}}_{{1}} {\text{x}}_{{1}}^{{2}} + \cdots + {\text{w}}_{{\text{n}}} {\text{x}}_{{\text{n}}}^{{2}} $$

In addition to using the square function, other nonlinear functions such as logarithmic, exponential and trigonometric functions can also be applied to transform the independent variables. This allows the model to adapt to more complex nonlinear relationships.

It is important to note that nonlinear extension can improve the fitting capability of the model and make it more suitable for nonlinear data. However, the resulting extended model may be more complex, less interpretable and have a risk of overfitting. Therefore, when performing nonlinear extension, a trade-off between the accuracy of model fitting and interpretability needs to be considered.

Data-driven models, such as regression-based approaches, have been widely used for air ozone prediction. Linear regression (LR) is a statistical modeling technique used to establish a linear relationship between a dependent variable and one or more independent variables. In air ozone prediction, LR models can be employed to identify correlations between ozone levels and relevant factors, such as temperature, humidity, wind speed and pollutant concentrations. Researchers have utilized various variables, including meteorological parameters, pollutant concentrations and emission data, to develop accurate prediction models. For example, Wei Zhao employed multiple linear regression to predict ozone levels based on boundary layer height, humidity, wind direction, surface solar radiation, total cloud cover and sea level pressure in Hong Kong^13^.

#### Neural networks

BPNN (Backpropagation Neural Networks) and RNN (Recurrent Neural Networks) are two commonly used artificial neural networks, respectively suitable for regression tasks and sequential data processing.

**Figure 1:**
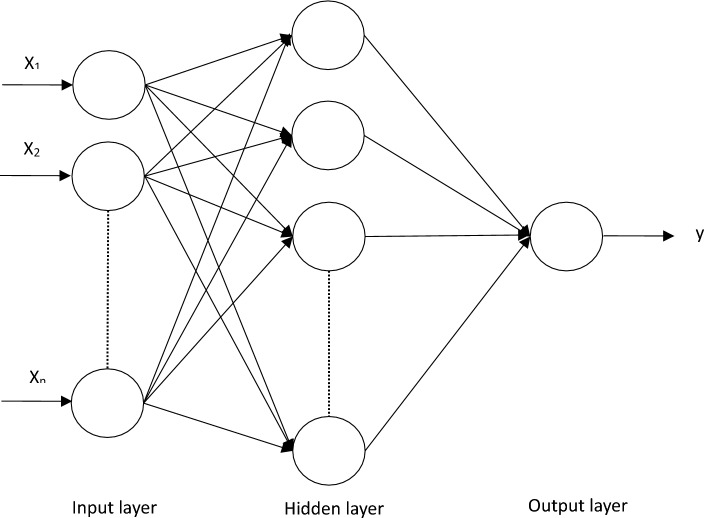
BPNN model.

BPNN utilizes the backpropagation algorithm to train the network by iteratively adjusting the weights and biases of the neurons to minimize the difference between the predicted and actual output,as shown in Fig. 1. This iterative process helps the model capture complex non-linear relationships between input and output variables, making it suitable for various regression problems.

**Figure 2:**
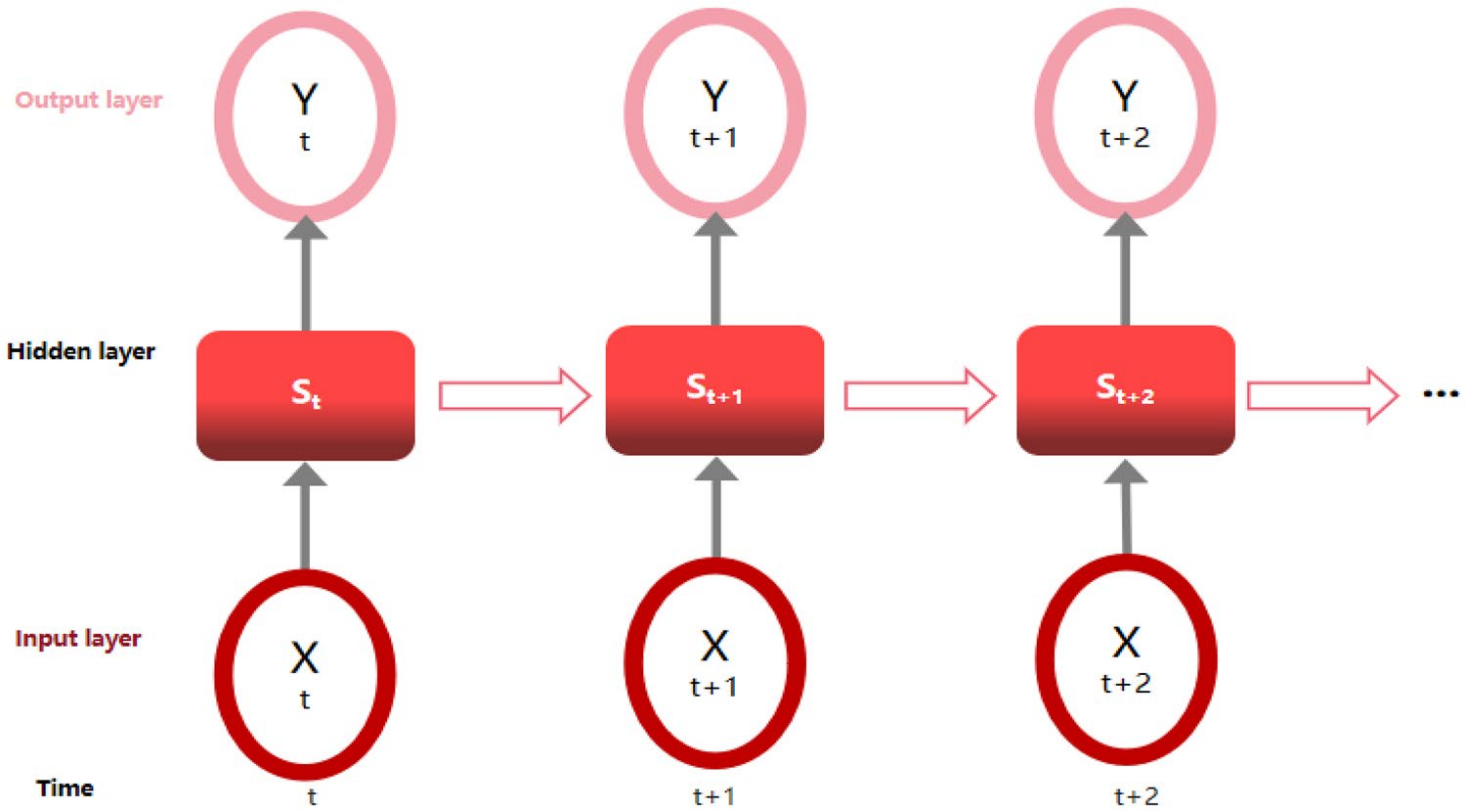
RNN model.

RNN is a type of neural networks designed to process sequential data, such as time series or text data. Unlike BPNN, RNN has a feedback mechanism that allows information to be carried forward through time loops, as shown in Fig. 2. This recurrent structure enables RNN to capture temporal dependencies and contextual information within the data. In regression tasks, RNN can model the sequence of input variables and predict the corresponding continuous output. They are particularly useful for problems where past inputs have a significant impact on current predictions.

Machine learning techniques have gained popularity in air ozone prediction due to their ability to capture complex relationships in data. Neural networks are computational models inspired by the structure and functioning of biological neural networks. These models consist of interconnected nodes (neurons) organized in layers and are trained using optimization algorithms to learn complex patterns in the data. For air ozone prediction, neural networks can capture nonlinear relationships between predictor variables and ozone concentrations.Neural networks, including BPNN and RNN, have been utilized for ozone prediction. RNN possesses feedback connections that allow information to flow between different time steps, making them ideal for time series analysis and prediction. In air ozone prediction, RNN can effectively capture temporal dependencies and patterns in ozone data.RNN, in particular, has shown promise in capturing temporal dependencies and patterns in ozone data^14,15^. Wang Dongsheng et al. developed an RNN model to predict hourly ozone concentrations in air quality monitoring stations in the Yangtze River Delta, China^16^.

#### Random forest regression

**Figure 3:**
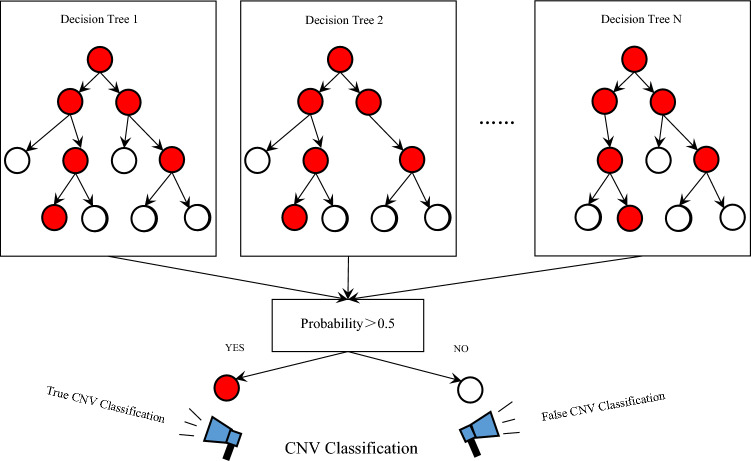
RFR model.

RFR (random forest regression) is an ensemble learning technique that combines the power of decision trees and randomness. It constructs a multitude of decision trees using random subsets of the training data and randomly selected subsets of the input variables. Each decision tree makes independent predictions and the final prediction is obtained by averaging the predictions of all the trees,as shown in Fig. 3. RFR handles both linear and non-linear relationships, effectively captures complex interactions between input variables and is robust against overfitting. It is particularly suitable for high-dimensional data with categorical and numerical features and performs well even in the presence of outliers and missing values.

Ensemble models, such as RFR (random forest regression) and gradient boosting, have also been applied for air ozone prediction^17,18^. RFR is an ensemble learning method that combines multiple decision trees to make predictions. Each decision tree is built using a random subset of features and the final prediction is determined by aggregating the predictions from individual trees. RFR is known for its robustness, ability to handle high-dimensional data and resistance to overfitting^19^. For instance, Massimo Stafoggia et al.^21^ used RFR to predict daily ozone concentrations in Sweden, considering various meteorological variables such as air temperature, cloud coverage, barometric pressure and snow albedo^20^.

### Applications of methods in environmental prediction

LR, NN and RFR have been widely employed in various environmental prediction tasks beyond air ozone prediction.

#### Water quality prediction

These methods have found applications in areas such as water quality prediction. LR, NN and RFR have been used to predict water quality parameters, including dissolved oxygen levels, pH and nutrient concentrations^21–23^.

#### Air pollutant concentration modeling

NN and RFR have been applied to forecast concentrations of air pollutants, such as particulate matter (PM) and nitrogen dioxide (NO_2_)^24,25^.

#### Environmental impact assessment

LR and NN have been applied for environmental impact assessment, such as global warming, human health, metal depletion, freshwater ecotoxicity, particulate matter formation and terrestrial acidification^26–28^.

These examples highlight the versatility and effectiveness of these modeling techniques in addressing a range of environmental prediction tasks.

### Performance in ozone prediction of prediction models

LR, NN and RFR are prediction models based on different principles and algorithms. LR predicts by fitting a linear relationship between input features and output variables. NN utilizes multi-layered neuron networks to establish nonlinear mapping relationships. RFR combines multiple decision tree models through ensemble learning to enhance prediction performance.

To accurately predict ozone concentrations and trends, various prediction methods have been employed.The performance of commonly used different prediction models in ozone prediction is compared as Table 1.

**Table 1:** Methods used in ozone concentrations prediction.

References	Variables/inputs	Targets/outputs	Performance	Model
^13^	Boundary layer height, humidity, wind direction, solar radiation, total cloud cover and sea level pressure, temperature	Surface ozone in Hong Kong	R^2^0.62	LR
^29^	Temperature, NO_2_, SO_2_, O_3_, PM_10_	Future ozone concentration for next three days in Malaysia	R^2^0.296996 RMSE0.01853	LR
^30^	Temperature, NO_2_, NO, wind velocity, relative humidity	Ozone concentration of Northern Portugal	R^2^0.7 RMSE29.5 μg/m^3^	LR
^30^	Temperature, NO_2_, NO, wind velocity, relative humidity	Ozone concentration of Northern Portugal	R^2^0.78 RMSE25.64 μg/m^3^	BPNN
^31^	Meteorological parameters, NO_2_	Ozone concentration of Nanjing	R^2^0.84 RMSE22.5	BPNN
^32^	Precipitation, barometric pressure relative humidity, sunshine duration temperature, wind speed	Ozone concentration of Jinan	R^2^0.8429 RMSE21.9290	BPNN
^15^	Temperature, dew point, relatively humidity, wind speed	Ozone concentration of Hangzhou	R^2^0.91 RMSE19.87	RNN
^9^	NO_X_, CO, PM_10/2.5_, VOC_S_, winds peed, temperature, humidity, radiation	Hourly ozone concentration in Shanghai	R^2^0.96 RMSE7.71	RNN
^18^	Temperature, dew point, relatively humidity, wind speed humidity, wind speed	Ozone Concentration of Hangzhou	R^2^0.85 RMSE27.64	RFR
^33^	Evaporation, temperature, relatively humidity, day of year, sunshine duration	Daily ambient ozone levels across China	R^2^0.69 RMSE26	RFR

LR is sensitive to linear relationships in data, making it suitable for predicting simple linear patterns.NN and RFR exhibit better adaptability to complex nonlinear relationships.

When it comes to air ozone prediction, LR assumes a linear relationship between the ozone concentration and predictor variables. Therefore, if the ozone concentration exhibits a clear linear trend and is influenced by straightforward factors such as meteorological or environmental variables, LR can provide accurate predictions.

However, in reality, ozone concentrations are often affected by complex nonlinear relationships, such as the interaction of multiple environmental factors or the impact of nonlinear pollution sources. In such cases, NN and RFR models can handle the complexity of the relationships more effectively.

NN is based on the principles of the biological nervous system, and they excel at capturing nonlinear patterns in the data. By learning the nonlinear features of the training data, NN can model the complex relationship between ozone concentration and various meteorological and environmental factors, providing more accurate predictions.

RFR, on the other hand, is an ensemble learning method that combines multiple decision trees. It can handle nonlinear relationships by creating an ensemble of trees that collectively capture the complex interactions between predictor variables and the ozone concentration. This ensemble approach allows RFR to provide more robust and accurate predictions in the presence of complex and noisy nonlinear relationships.

### Comparison of prediction models

LR is suitable for air ozone prediction when the relationship between predictors and ozone concentration is linear and straightforward. However, when the relationship becomes more complex and nonlinear, NN and RFR are better equipped to capture and model these complexities, offering more accurate and reliable predictions. The choice of the appropriate model ultimately depends on the specific characteristics of the data and the requirements of the ozone prediction task.

NN, with their ability to capture intricate nonlinear patterns, is particularly suitable for large datasets and complex problems. The architecture of neural networks, consisting of multiple layers of interconnected nodes (neurons), allows them to learn and extract complex features from the data. This enables NN to model the complex relationships between the ozone concentration and various meteorological and environmental factors, leading to higher prediction accuracy.

BPNN and RNN are two commonly used neural network models for handling different types of data and problems. They also have different performance when it comes to time series prediction.

BPNN is a widely used feedforward neural network model, primarily designed to address classification and regression problems. It can learn and capture nonlinear relationships and trends in time series data for accurate predictions. However, BPNN may have difficulties with long-term dependencies in time series data, as its training process relies only on the current input and previous feedback. When there are long time delays or complex dependencies between time series data, BPNN may struggle to capture these patterns accurately, leading to decreased accuracy.

RNN and BPNN have distinct characteristics in time series prediction tasks. RNN is well-suited for modeling sequential dependencies and can handle variable-length time series data. It excels in capturing long-term dependencies and complex relationships through its recurrent connections and memory-like components.On the other hand, BPNN lacks explicit memory of past information and is less suitable for modeling sequential dependencies. However, BPNN can still be used for time series prediction by converting temporal data into a fixed-size input format, such as using month or date variables. In terms of performance, RNN tends to achieve better accuracy, especially in tasks involving long-range dependencies, but it requires more training data and may suffer from overfitting. BPNN can perform reasonably well with less training data.

LR offers fast computation with shorter model training and prediction time. NN and RFR models require more computational resources and time due to their larger model complexity.

## Methodology

### Data collection and preprocessing

The data used in this study consists of air pollution and meteorological records from a city in Sichuan Province, China, with a permanent population of over 20 million, spanning the past 9 years from 2014 to 2022. The variables included in the dataset are O_3_ (24-h average), PM_2.5_ (24-h average), PM_10_ (24-h average), SO_2_ (24-h average), NO_2_ (24-h average), CO (24-h average), daily average temperature, daily average wind speed, daily sunshine duration, daily mean temperature and month.

The data collection process involved obtaining daily air pollution and meteorological data from reliable sources. These data were collected ensuring a comprehensive representation of different seasonal and temporal patterns.The air pollution and meteorological data were merged into a single dataset based on the common timestamp of each daily observation. This integration facilitated the modeling process by providing a consolidated view of all relevant variables.

The dataset was split into a training set and a testing set. Sample 1–2773 were used as training set, while the remaining samples (sample 2774–3138), representing the period from January 1, 2022 to December 31, 2022, were set aside as testing set. This partitioning allowed us to assess the performance of the soft sensor models on unseen data.

To ensure that all variables have a similar range and distribution, data scaling techniques such as normalization or standardization were applied. This step is important for models that are sensitive to the scale and variance of input features.

Finally, the preprocessed data was carefully verified to ensure its integrity and suitability for the soft sensor modeling. Any inconsistencies or errors were addressed before proceeding to model development.

By following these data collection and preprocessing steps, we prepared a high-quality dataset for the subsequent modeling analysis. This dataset incorporated both air pollution and meteorological variables, allowing us to develop accurate soft sensor models for air ozone prediction.

### Feature selection and engineering

Feature selection techniques, including Pearson correlation coefficient analysis, were applied to identify the most relevant variables for ozone prediction. This step aimed to reduce dataset dimensionality, improve model interpretability and ensure that only the most influential features were incorporated in the modeling process, thus mitigating the risk of overfitting. By assessing the strength and direction of relationships between variables, the Pearson correlation coefficient analysis helped identify variables that significantly correlated with ozone levels. This allowed us to concentrate on the most informative predictors, leading to more accurate and interpretable soft sensor models while reducing the risk of overfitting.

Pearson correlation coefficient is a statistical measure used to determine the strength and direction of the linear relationship between two variables. Essentials of application of Pearson correlation coefficient in variables correlation ranking are: (1) Pearson correlation coefficient is commonly used in multiple regression analysis to select the most significant independent variables by calculating the correlation coefficients between each independent variable; (2) The correlation coefficient ranges from − 1 to 1, and the larger the absolute value, the stronger the correlation; (3) When the correlation coefficient value is close to 0, it indicates that the correlation between the two variables is very weak and they can be considered independent.

**Figure 4:**
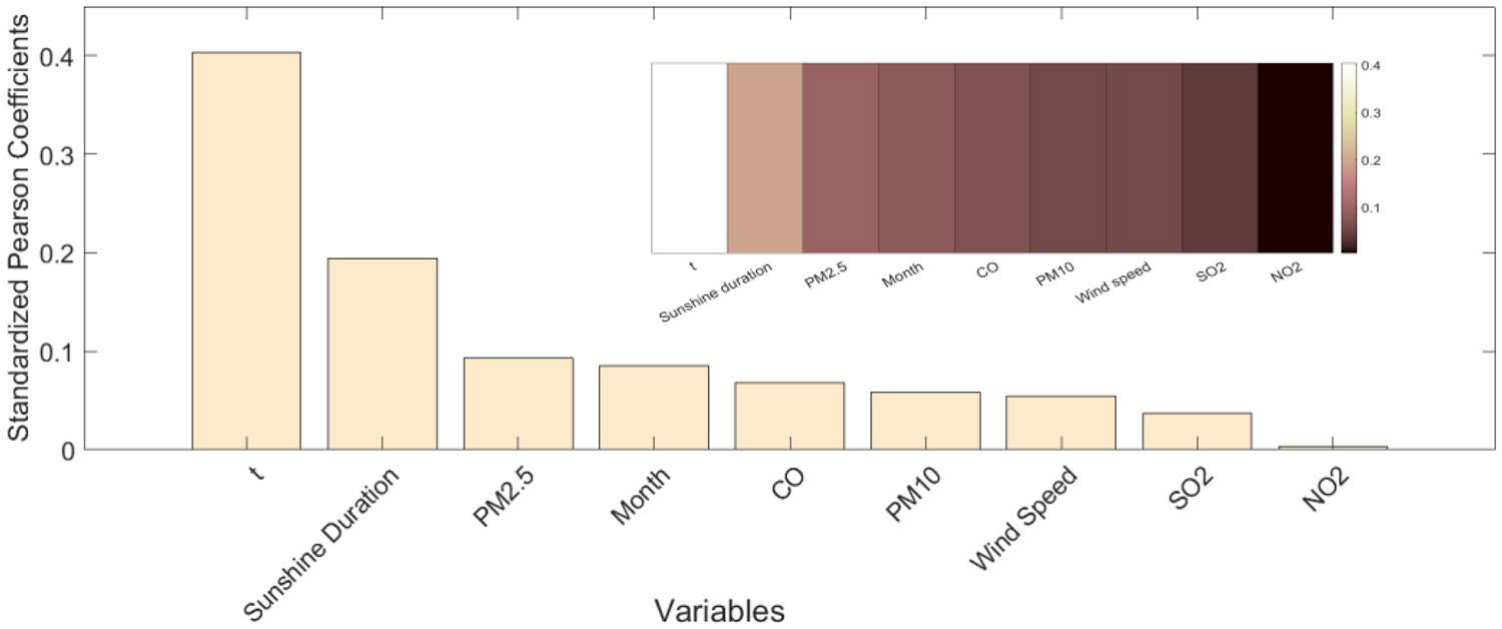
Ranking of variable importance.

In order to prevent the occurrence of invalid variables, avoid overfitting and improve the training performance of the model, any variable with a normalized Pearson correlation coefficient value, that is regarded as the normalized score of variable importance, less than 0. 01 was removed. The resulting normalized score of variable importance ordering diagram shows the nine factors affecting ozone concentration (Fig. 4). It was found that temperature had the greatest influence on ozone concentration, followed by sunshine duration, PM_2.5_, month, CO, PM_10_, wind speed, SO_2_ and NO_2_.

**Figure 5:**
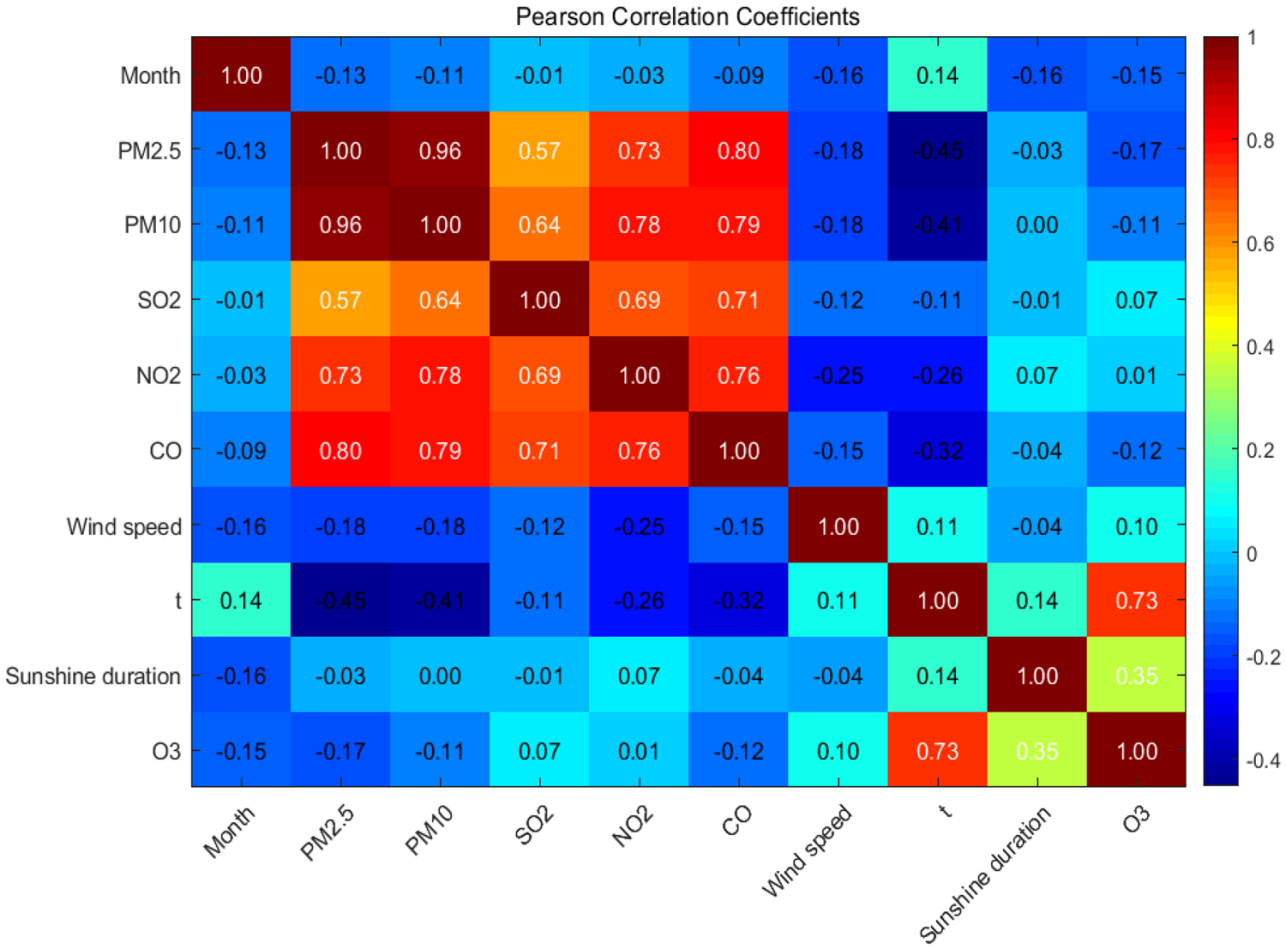
Pearson correlation between variables.

Calculating and analyzing the Pearson correlation coefficient between variables is done to detect the presence of high correlation or multicollinearity among the independent variables (Fig. 5). Multicollinearity can have an impact on the interpretability and stability of the model, as well as affect the accurate assessment of the coefficients and statistical significance of the independent variables. With multicollinearity, the effects of the independent variables become difficult to independently explain, and it becomes challenging for the model to determine the unique contribution of each independent variable towards the dependent variable. Recognizing these issues and taking appropriate measures to address multicollinearity can improve the quality and reliability of the model.

For example, the Pearson correlation coefficient between PM_10_ and PM_2.5_ is + 0.96, indicating a high degree of correlation. This can be well explained by the common meaning and measurement methods of these two variables.

**Figure 6:**
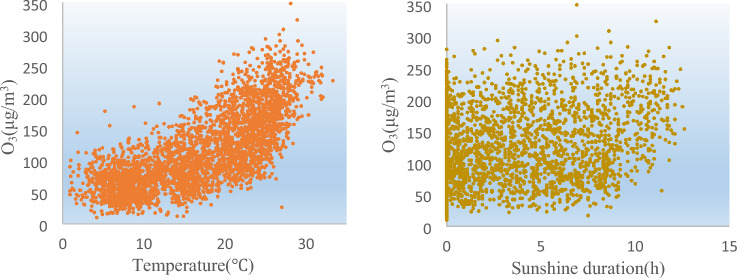
Relationship between meteorological conditions and ozone concentration.

Based on the ranking of variable importance scores (Fig. 4), it is evident that meteorology-related variables hold the top two positions. Therefore, it can be concluded that meteorology-related variables play a dominant role in the data analysis within the range of the nine variables under consideration. Similarly, the relationship diagrams between the aforementioned two and ozone are provided (Fig. 6).

Studies^34–36^ have highlighted the relationship between temperature and ozone concentration, providing valuable insights into the complex dynamics involved. The warming of the lower atmosphere can greatly influence the concentration of ozone due to its impact on photochemical reactions.

At higher temperatures, the molecular collisions and reactions involved in the production and destruction of ozone become more frequent and energetic. This enhanced molecular activity promotes the production of reactive species like NO_x_ and VOC_s_. These species play a crucial role in the ozone generation process. The positive correlation between ozone and temperature can be explained by enhanced photochemical reaction: at high temperatures, the intensity of solar radiation increases, which facilitates photochemical reactions between NO_x_ and VOC_s_ in the atmosphere, resulting in the formation of more ozone.

Turning to the influence of sunlight duration on ozone concentration, it is widely recognized that prolonged exposure to sunlight provides more energy for photochemical reactions to occur. Sunlight, particularly in the ultraviolet wavelength range, initiates a series of complex photochemical reactions that ultimately lead to ozone formation.

The primary photochemical reaction involved in ozone generation is the dissociation of nitrogen dioxide (NO_2_) into individual oxygen atoms (O). These oxygen atoms then react with O_2_ molecules to form ozone (O_3_). Longer durations of sunlight exposure increase the availability of UV radiation required for the dissociation of nitrogen dioxide, leading to a higher ozone production rate.

In theory, there is a correlation between air ozone and NO_x_, as NO_x_ is a precursor to the formation of ozone. However, in this sample set, Pearson correlation coefficient analysis found no correlation between ozone and NO_2_, which is inconsistent with theory or common perception. The reasons for this result may be: (1) time lag effect: the correlation between ozone and nitrogen dioxide may be affected by time lag effect. Due to the time difference between the formation and transformation processes of ozone and nitrogen dioxide in the atmosphere, there may be a lack of correlation between the measured data at a certain point in time. (2) Composition randomness: there is a certain degree of randomness in the ratio between NO and NO_2_ in NO_X_, which may affect the Pearson correlation coefficient. The Pearson correlation coefficient is an indicator that measures the degree of linear correlation between two variables. If one of the variables (such as the ratio of NO to NO_2_ in NO_X_) has significant randomness, this may lead to a low correlation coefficient or lack of statistical significance in the calculation results. Therefore, when conducting correlation analysis, it is necessary to consider the range of variation in the ratio of NO to NO_2_ in NO_X_ and the stability of the data to obtain more accurate results. Unfortunately, there is no separate statistics of NO_X_ concentration in the samples of this investigation, because NO_2_ is a required test item in the local area, while NO_X_ is an optional test item.

In a regression model, the combination of input variables has a significant impact on the model's performance and predictive ability. Here are two key factors to consider:

- Dimension and quantity.
  The dimension refers to the number of input features, while the quantity represents the number of different types of input features. Having higher dimensions and quantities can provide more information and variations, which in turn enhance the model's expressive power and fitting ability. However, it is essential to exercise caution when selecting the appropriate dimension and quantity to avoid issues such as overfitting.
- Feature selection.
  Utilizing feature selection methods helps identify input variables with high predictive power for the target variable. By excluding irrelevant or redundant features, the model's complexity can be reduced, leading to improved generalizability. Pearson coefficient is a commonly used metric to assess the linear correlation between input variables and the target variable. Higher Pearson coefficients indicate stronger linear relationships, thus providing guidance for feature selection. Feature ranking is an effective technique that ranks features based on their correlation with the target variable. By calculating the Pearson coefficient, the strength of the linear relationship between variables can be determined and used to rank the features. Based on this ranking, variables with higher Pearson coefficients can be selected as input features to enhance the model's predictive ability. It's also possible to construct multiple models with different input variable combinations based on feature ranking (Table 2). By comparing the performance of these models, the optimal input variable combination can be determined for building the regression model.

**Table 2:** Input combinations based on ranking of variable importance.

Variable set	t	SD	PM_2.5_	Month	CO	PM_10_	WS	SO_2_	NO_2_
A	+	+	+	+	+	+	+	+	+
B	+	+	+	+	+	+	+	+	
C	+	+	+	+	+	+	+		
D	+	+	+	+	+	+			
E	+	+	+	+	+				
F	+	+	+	+					
G	+	+	+						
H	+	+							
I	+								

*SD* sunshine duration, *WS* wind speed.
  If input variables have different scales or units, feature scaling becomes crucial. Standardization and normalization are common techniques used to ensure that all input variables have similar scales, avoiding the undesirable influence of different scales on the model during the training process.
  Overall, selecting the appropriate combination of input variables requires a comprehensive consideration of the data's characteristics, problem complexity, and model requirements. By selecting and processing input variables thoughtfully, we can enhance the model's accuracy and generalization ability, while carefully avoiding overfitting. Consequently, when constructing a regression model, it's essential to pay close attention to the impact of input variable selection and feature processing.The variable set is determined based on the importance determined by the Pearson correlation coefficient. The variables are ranked by their importance, and the top 9–1 variables are selected for combination (Table 2).

## Application

### Models

Three types of inferential estimation models were examined, namely LR (linear regression), NN (neural networks) and RFR (random forest regression). Two LR models were applied, R_ML_ (MLR without nonlinear extension) and R_MLNE_ (MLR with nonlinear extension).

Different NN models can have different numbers of neurons^37^. For example, a BPNN model can be designed with the number of neurons equal to 1 or 2 times the number of input variables(NN_BP[X]_ and NN_BP[2X]_).

The RNN model can be designed with two variations^38^: one that utilizes the first 30 time steps (NN_R[Y]_) and another that utilizes the first 15 time steps (NN_R[0.5Y]_).

The RFR model can be designed with a type^39^ that has 100 trees or a type that has 200 trees (RFR_100_ and RFR_200_).The comparison of the eight regression models is shown in Table 3.

**Table 3:** Comparison of models.

Model type	Model name	Description	Differences with the other similar model
Linear regression	R_ML_	Multiple Linear Regression model with multiple independent variables, assuming a linear relationship between the dependent variable and the independent variables	Multiple Linear Regression model with nonlinear terms has the advantage of allowing for a more complex relationship between the dependent variable and the independent variables, which can improve the fitting capability of the model, especially for nonlinear data
	R_MLNE_	Multiple Linear Regression model with nonlinear terms, allowing for a more complex relationship between the dependent variable and the independent variables. Differs from R_ML_ in the inclusion of nonlinear terms	
Neural network	NN_BP[X]_	An artificial neural network model trained using the backpropagation algorithm with 1 times the number of input variables in the hidden layer(s). Differs from NN_BP[2X]_ in the number of neurons in the hidden layer(s)	The main difference between these two models lies in their complexity and potential learning capability. NN_BP[2X]_ has a higher number of neurons, which increases the model's capacity to learn more complex relationships between the input and output variables. This can lead to better fitting results and more accurate predictions.A higher number of neurons also increases the risk of overfitting, as the model may become too complex and fit the noise in the training data
	NN_BP[2X]_	An artificial neural network model similar to NN_BP[X]_ but with twice as many neurons in the hidden layer(s)	
Recurrent neural network	NN_R[Y]_	A neural network model that can process sequential data, using the first 30 time steps to make predictions. Differs from NN_R[0.5Y]_ in the number of time steps used for prediction	The main difference between these two models lies in the amount of historical information they consider when making predictions. NN_R[Y]_ takes into account a longer sequence of past data, which may provide more context and improve the model's ability to capture temporal patterns and trends. Using more time steps also increases the computational complexity of the model and may require more data to train effectively
	NN_R[0.5Y]_	A neural network model similar to NN_R[Y]_ but uses the first 15 time steps for prediction	
Random forest regression	RFR_100_	An ensemble learning model that combines multiple decision trees for regression prediction, using 100 decision trees. Differs from RFR_200_ in the number of decision trees used	The increased number of decision trees in RFR200 generally leads to a more complex model, which can capture more subtle patterns in the data and potentially result in more accurate predictions. However, this comes at the cost of increased computational complexity and a higher risk of overfitting, particularly if the dataset is small
	RFR_200_	An ensemble learning model similar to RFR_100_ but uses 200 decision trees for regression prediction	

In total, 72 soft sensors (i.e., a model applied to a variable set) were analysed. These soft sensors consisted of eight models with nine identified variable sets using 1–9 input variables (Table 2).

### Assessment of soft sensor model

The effectiveness of the soft sensor model was assessed across five criterion. The standard value of ozone was set at 160 μg/m^3^.Ozone standard value can vary due to local regulations. The assessment criterion are listed in Table 4.

**Table 4:** Criterion of assessment.

Criterion	Description	Practical application
R^2^	Referred to as the coefficient of determination, it is an indicator of the strength of the relationship between variables	Measures the strength of the relationship between predicted trend and actual trend
RMSE	Root Mean Square Error (RMSE) is another widely used statistical metric to evaluate the performance of a model. It measures the square root of the average of the squared differences between the predicted and actual values. Similar to MSE, a lower RMSE value indicates a higher level of accuracy in prediction	Measures the average accuracy of the predicted trend against the actual trend
MAE	Mean Absolute Error (MAE) is a commonly used statistical metric to assess the performance of a model. It calculates the average of the absolute differences between the predicted and actual values. MAE provides a measure of the average magnitude of the errors, disregarding their direction. Similar to MSE and RMSE, a lower MAE value indicates a higher level of accuracy in prediction	Measures the average accuracy of the predicted values compared to the actual values. Instead of focusing solely on the differences between predicted and actual values, MAE calculates the average magnitude of these differences. It provides a meaningful measure of the average prediction error, regardless of the direction of the errors
Variable utilization	Variable utilization refers to the number of input variables used by each soft sensor, ranging from 1 to 9	Represents the amount of data needed, which indirectly reflects the amount of pre-foundation work
Accuracy	When the measured ozone concentration is above the local standard value, if the prediction is valid and significant, that means the predicted value is greater than the standard value,the accuracy meets the requirement.Accuracy(%) is equal to the number of successful predictions divided by the number of occurrences where the measured values exceeded the threshold	Indicates the accuracy at the threshold(standard value). The criterion of accuracy expresses the concern and attention to the predictive ability of ozone concentration exceeding the standard. As an example, when the measured value of air ozone concentration is 210 μg/m^3^, which exceeds the local ambient air quality standard (ozone, 160 μg/m^3^), if the predicted value is greater than 160 μg/m^3^, then it indicates that the prediction of the fact that the standard has been exceeded has been successful; otherwise, it indicates a prediction failure. The accuracy is calculated by dividing the number of successful predictions in the test set by the number of days in the test set with all the metrics exceeding the threshold

Different criterion have different levels of importance. To compare these criterion, they need to be quantified and assigned weights. According to the consultation with environmental monitoring and air pollution control engineers, the following criterion weights have been obtained (Table 5).

**Table 5:** Weight of criterion.

Criterion	Ranking attributes	Functions	Weight
Accuracy	The higher the Accuracy value, the better the performance and the higher the ranking value	The higher the Accuracy value, indicating a better prediction performance in terms of correctly identifying instances where the ozone concentration exceeds the local standard value. This criterion emphasizes the importance of accurately predicting ozone concentration exceedances, providing a measure of the model's ability to capture such events	10
R^2^	The higher the R^2^ value, the better the performance and the higher the ranking value	The higher the R^2^ value, indicating a stronger relationship between variables and a better fit of the model to the data. This practical application provides insight into the model's ability to capture variations in the data	4
RMSE	The lower the RMSE value, the better the performance and the higher the ranking value	This criterion provides a comprehensive evaluation of the model's performance, considering both the magnitude and direction of the errors	3
MAE	The lower the MAE value, the better the performance and the higher the ranking value	MAE provides a comprehensive measure of the average prediction error, considering both the magnitude and direction of the errors. This criterion effectively evaluates the model's ability to minimize the overall prediction error, offering insight into its predictive performance	2
Variable utilization	The lower the variable utilization value, the better the performance and the higher the ranking value	The variable utilization indirectly reflects the amount of pre-foundation work needed, such as data acquisition, feature engineering, and data cleaning. This attribute offers valuable information about the potential complexity and resources needed for the implementation of each soft sensor	1

### Modeling process

According to the principle of prediction model and evaluation methods,the modeling process is divided into four steps as follows: (1) collection of sample data; (2) determination and ranking of the importance of features; (3) construction of variables database; (4) prediction model applied in practice; (5) evaluation of soft sensors.

**Figure 7:**
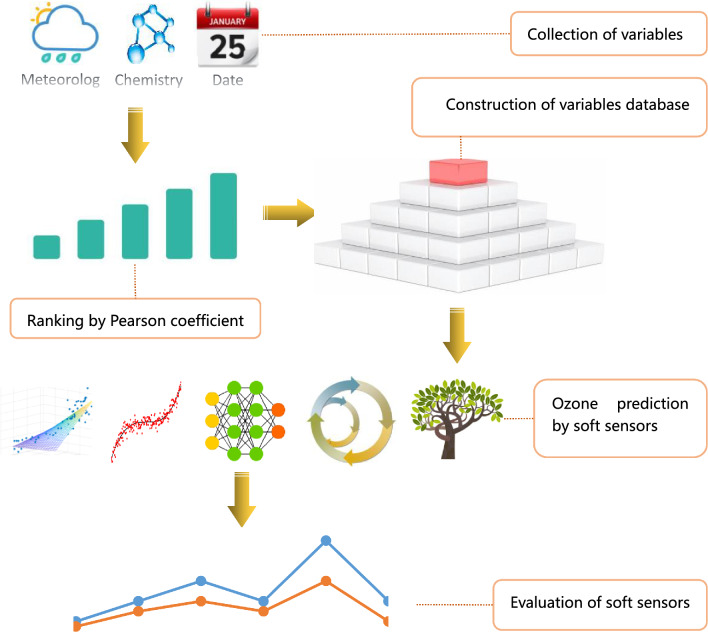
The technical balance between the variables and prediction model.

Based on the operational process data, soft sensor models were utilized to develop the ozone prediction model, which is illustrated in Fig. 7.

## Results and analysis

### Results of LR

Two regression models were assessed, R_ML_ and R_MLNE_. Detailed results for each model are displayed in Table 6, respectively. An overview of these results confirms that R_MLNE_ outperformed R_ML_ in terms of accuracy, as it consistently achieved higher R^2^ and lower error results. This improvement in accuracy leads to more reliable ozone concentration predictions.

**Table 6:** Results of LR.

Sensor	R^2^	RMSE	MAE	Variable utilization	Accuracy (%)
R_MLA_	0.7271	29.94	24.4891	9	59.820
R_MLB_	0.7262	29.99	24.1181	8	60.680
R_MLC_	0.7296	29.81	23.7566	7	60.680
R_MLD_	0.7297	29.8	23.7594	6	60.680
R_MLE_	0.7152	30.59	25	5	62.390
R_MLF_	0.7119	30.77	24.616	4	60.680
R_MLG_	0.7211	30.27	24.4848	3	55.550
R_MLH_	0.6851	32.17	26.3866	2	52.990
R_MLI_	0.6928	31.77	26.2531	1	49.570
R_MLNEA_	0.7642	27.83	22.7889	9	57.260
R_MLNEB_	0.7318	29.69	23.802	8	60.680
R_MLNEC_	0.7334	29.6	23.7532	7	61.530
R_MLNED_	0.7337	29.58	23.7448	6	58.970
R_MLNEE_	0.7254	30.04	24.3147	5	59.830
R_MLNEF_	0.7278	29.91	24.2875	4	60.680
R_MLNEG_	0.7247	30.07	24.0526	3	63.250
R_MLNEH_	0.7156	30.57	24.7728	2	63.250
R_MLNEI_	0.7228	30.18	24.5716	1	47.000

LR with nonlinear expansion is a machine learning method that enhances the flexibility and expressive power of a model by applying nonlinear transformations to input features. In traditional linear regression, it is assumed that there is a linear relationship between the features and the target variable. However, in real-world problems, many factors do not satisfy the linear assumption. LR with nonlinear expansion introduces nonlinear functions to map the original features, enabling the model to capture more complex relationships. This approach improves the predictive accuracy of the model and is suitable for modeling nonlinear relationships.

Sensor R_MLD_ and R_MLNEC_ perform best in their respective model categories, as shown in Tables 7 and 8, which means that having more variables in a variable set does not necessarily result in better sensor performance. Overfitting should be particularly considered, so the variable set should be carefully determined.

**Table 7:** Ranking values of R_ML_.

Sensor	Ranking values in the following criterion					Weighted ranking values
	R^2^	RMSE	MAE	Variable utilization	Accuracy	
R_MLA_	7	7	5	1	4	100
R_MLB_	6	6	7	2	5	108
R_MLC_	8	8	9	3	5	127
R_MLD_	9	9	8	4	5	133
R_MLE_	4	4	4	5	9	131
R_MLF_	3	3	3	6	5	83
R_MLG_	5	5	6	7	3	84
R_MLH_	1	1	1	8	2	37
R_MLI_	2	2	2	9	1	37

**Table 8:** Ranking values of R_MLNE_.

Sensor	Ranking values in the following criterion					Weighted ranking values
	R^2^	RMSE	MAE	Variable utilization	Accuracy	
R_MLNEA_	9	9	9	1	2	102
R_MLNEB_	6	6	6	2	5	106
R_MLNEC_	7	7	7	3	7	136
R_MLNED_	8	8	8	4	3	106
R_MLNEE_	4	4	3	5	4	79
R_MLNEF_	5	5	4	6	5	99
R_MLNEG_	3	3	5	7	8	118
R_MLNEH_	1	1	1	8	8	97
R_MLNEI_	2	2	2	9	1	37

### Results of NN

Four regression models were assessed, NN_BP[X]_, NN_BP[2X]_, NN_R[Y]_ and NN_R[0.5Y]_. Detailed results for each model are displayed in Tables 9 and 10, respectively. An overview of these results confirms that NN_R_ outperformed NN_BP_ in terms of accuracy, as it consistently achieved higher R^2^ and accuracy. This improvement leads to more reliable ozone concentration predictions.

**Table 9:** Results of NN_BP_.

Sensor	R^2^	RMSE	MAE	Variable utilization	Accuracy (%)
NN_BP[X]A_	0.87274	25.7923	20.189	9	76.289
NN_BP[X]B_	0.8758	27.0951	20.8078	8	79.381
NN_BP[X]C_	0.8728	26.9391	20.88	7	83.505
NN_BP[X]D_	0.86533	24.7628	19.0255	6	79.381
NN_BP[X]E_	0.8722	24.1676	18.8387	5	81.443
NN_BP[X]F_	0.86969	25.774	19.9231	4	76.289
NN_BP[X]G_	0.81766	25.9039	20.5584	3	71.134
NN_BP[X]H_	0.76605	30.08	24.2408	2	70.103
NN_BP[X]I_	0.7392	31.4552	25.1164	1	63.918
NN_BP[2X]A_	0.8799	25.2692	19.8742	9	76.289
NN_BP[2X]B_	0.88129	26.3355	20.7613	8	78.351
NN_BP[2X]C_	0.84936	26.3093	20.8789	7	70.103
NN_BP[2X]D_	0.87687	28.0203	19.916	6	79.381
NN_BP[2X]E_	0.86211	25.6323	19.9623	5	84.536
NN_BP[2X]F_	0.85464	28.1521	21.6367	4	77.32
NN_BP[2X]G_	0.81043	25.4879	20.2439	3	70.103
NN_BP[2X]H_	0.77477	31.7843	25.8355	2	70.103
NN_BP[2X]I_	0.73017	34.3527	27.6884	1	59.794

**Table 10:** Results of NN_R_.

Sensor	R^2^	RMSE	MAE	Variable utilization	Accuracy (%)
NN_R[Y]A_	0.90	25.8317	20.0169	9	78.351
NN_R[Y]B_	0.9001	24.2706	18.709	8	78.351
NN_R[Y]C_	0.8902	24.9123	19.1583	7	81.443
NN_R[Y]D_	0.8962	25.7434	20.013	6	80.412
NN_R[Y]E_	0.8838	26.0779	19.6821	5	83.505
NN_R[Y]F_	0.8530	27.5419	21.2768	4	80.412
NN_R[Y]G_	0.8498	26.6071	21.2003	3	71.134
NN_R[Y]H_	0.7928	30.5566	24.5275	2	70.103
NN_R[Y]I_	0.7370	33.3163	26.7145	1	59.794
NN_R[0.5Y]A_	0.867	26.6056	20.8383	9	78.351
NN_R[0.5Y]B_	0.8888	25.1199	19.8288	8	80.412
NN_R[0.5Y]C_	0.8808	26.4318	20.2491	7	80.412
NN_R[0.5Y]D_	0.8566	25.3612	19.6895	6	78.351
NN_R[0.5Y]E_	0.8802	27.0095	21.2078	5	82.474
NN_R[0.5Y]F_	0.8677	26.2297	19.9844	4	79.381
NN_R[0.5Y]G_	0.8436	26.1772	20.7957	3	70.103
NN_R[0.5Y]H_	0.8268	30.5001	24.5668	2	68.041
NN_R[0.5Y]I_	0.7323	32.0173	25.6736	1	65.979

RNN is a type of neural network architecture that exhibits strong capabilities in handling sequential data. Unlike traditional BPNN, RNN introduces recurrent connections, allowing information to be propagated within the network.

The key characteristic of RNN is its memory capability, enabling it to process input sequences of arbitrary length while considering the context information. By incorporating recurrent connections, RNN takes the previous time step's output as the current time step's input, allowing the network to model each element in the sequence and utilize past information to influence future outputs. This memory capability makes RNN highly effective in tasks involving time series and more.

In an RNN model, input delays refer to the range of delays in which the network receives input signals. It defines how many previous time steps the network considers at a given time step.

The choice of delay range depends on the nature of the problem and the temporal dependencies in the data. Longer delay ranges can help the network capture longer-term dependencies but also increase model complexity and computational costs^40^. Shorter delay ranges may limit the network's ability to model longer-term dependencies.

In practice, selecting the appropriate input delays requires experimentation and tuning to find the optimal delay range for achieving the best performance and contextual modeling capability on a given task.

Upon observation of BPNN results, NN_BP[2X]_ does not outperform NN_BP[X]_ in terms of performance.Setting the number of neurons to be equal to the input variables is a common practice, especially for relatively simple tasks and datasets. Such a setting often provides sufficient model capacity to learn and represent the features of the input data.When the number of input variables is small or simple, setting the number of neurons to be equal to the input variables maintains a relatively concise model that can effectively handle task requirements. This setting also helps reduce computational and memory costs, making the model training and inference processes more efficient.

The number of neurons is not necessarily the more, the better. The appropriate number of neurons depends on the specific problem and the structure of the neural network.

Increasing the number of neurons can enhance the model's expressive power and learning capacity, enabling it to better fit complex data patterns. Especially for large-scale and high-dimensional problems, appropriately increasing the number of neurons may improve the performance of the model.

However, having too many neurons can also lead to some issues. Firstly, it increases the complexity and computational load of the model, slowing down the training and inference process. Additionally, if there are too many neurons, it may result in overfitting, where the model excessively adapts to the training data and performs poorly on unseen data.

Therefore, when designing a neural network, it is important to determine the appropriate number of neurons based on the characteristics of the specific task and dataset. This often involves experimentation and optimization to find the optimal balance and achieve good model performance.

Upon observation of RNN results, NN_R[Y]_ outperformed NN_R[0.5Y]_ in terms of performance.Although this advantage may not be significant, it is still meaningful as it suggests that longer memory leads to better predictive performance.While broader memory yields better results, it may not necessarily be the optimal choice due to its higher computational requirements and the need for increased processing power.

Sensor NN_BP[X]E_, NN_BP[2X]E_, NN_R[Y]C_ and NN_R[0.5Y]B_ perform best in their respective model categories as shown in Tables 11, 12, 13 and 14.

**Table 11:** Ranking values of NN_BP[X]_.

Sensor	Ranking values in the following criterion					Weighted ranking values
	R^2^	RMSE	MAE	Variable utilization	Accuracy	
NN_BP[X]A_	6	6	6	1	4	95
NN_BP[X]B_	8	3	4	2	6	111
NN_BP[X]C_	7	4	3	3	9	139
NN_BP[X]D_	4	9	9	4	6	125
NN_BP[X]E_	9	8	8	5	8	161
NN_BP[X]F_	5	7	7	6	4	101
NN_BP[X]G_	3	5	5	7	3	74
NN_BP[X]H_	2	2	2	8	2	46
NN_BP[X]I_	1	1	1	9	1	28

**Table 12:** Ranking values of NN_BP[2X]_.

Sensor	Ranking values in the following criterion					Weighted ranking values
	R^2^	RMSE	MAE	Variable utilization	Accuracy	
NN_BP[2X]A_	8	9	9	1	5	128
NN_BP[2X]B_	9	5	5	2	7	133
NN_BP[2X]C_	4	6	4	3	2	65
NN_BP[2X]D_	7	4	8	4	8	140
NN_BP[2X]E_	6	7	7	5	9	154
NN_BP[2X]F_	5	3	3	6	6	101
NN_BP[2X]G_	3	8	6	7	2	75
NN_BP[2X]H_	2	2	2	8	2	46
NN_BP[2X]I_	1	1	1	9	1	28

**Table 13:** Ranking values of NN_R[Y]_.

Sensor	Ranking values in the following criterion					Weighted ranking values
	R2	RMSE	MAE	Variable utilization	Accuracy	
NN_R[Y]A_	8	6	5	1	4	101
NN_R[Y]B_	9	9	9	2	4	123
NN_R[Y]C_	6	8	8	3	8	147
NN_R[Y]D_	7	7	6	4	6	125
NN_R[Y]E_	5	5	7	5	9	144
NN_R[Y]F_	4	3	3	6	6	97
NN_R[Y]G_	3	4	4	7	3	69
NN_R[Y]H_	2	2	2	8	2	46
NN_R[Y]I_	1	1	1	9	1	28

**Table 14:** Ranking values of NN_R[0.5Y]_.

Sensor	Ranking values in the following criterion					Weighted ranking values
	R2	RMSE	MAE	Variable utilization	Accuracy	
NN_R[0.5Y]A_	5	4	4	1	4	81
NN_R[0.5Y]B_	9	9	8	2	7	151
NN_R[0.5Y]C_	8	5	6	3	7	132
NN_R[0.5Y]D_	4	8	9	4	4	102
NN_R[0.5Y]E_	7	3	3	5	9	138
NN_R[0.5Y]F_	6	6	7	6	6	122
NN_R[0.5Y]G_	3	7	5	7	3	80
NN_R[0.5Y]H_	2	2	2	8	2	46
NN_R[0.5Y]I_	1	1	1	9	1	28

### Results of RFR

Two RFR models were assessed, RFR_100_ and RFR_200_. Detailed results for each model are displayed in Table 15, respectively. Sensor RFR_100D_ and RFR_200F_ perform best in their respective model categories as shown in Tables 16 and 17. An overview of these results confirms that performance distinction between RFR_200_ and RFR_100_ in terms of criterion is not significant. Especially in terms of the most important criterion, accuracy, their results are consistent.

**Table 15:** Results of RFR.

Sensor	R^2^	RMSE	MAE	Variable utilization	Accuracy (%)
RFR_100A_	0.8215	24.9195	19.6193	9	73.196
RFR_100B_	0.8215	24.3742	19.1659	8	75.258
RFR_100C_	0.8176	24.5416	19.2376	7	76.289
RFR_100D_	0.8242	24.4918	19.2901	6	76.289
RFR_100E_	0.808	25.0466	19.7699	5	78.351
RFR_100F_	0.8023	25.2306	19.8128	4	81.443
RFR_100G_	0.7584	28.7701	23.2987	3	69.072
RFR_100H_	0.6768	32.6854	26.3009	2	67.010
RFR_100I_	0.7003	33.7101	27.3665	1	63.918
RFR_200A_	0.8254	24.889	19.5336	9	75.258
RFR_200B_	0.8273	22.9189	19.6088	8	74.227
RFR_200C_	0.823	24.3381	19.1967	7	78.351
RFR_200D_	0.821	24.4174	19.3058	6	78.351
RFR_200E_	0.8126	25.1275	19.7744	5	77.320
RFR_200F_	0.8508	25.3318	19.9433	4	79.381
RFR_200G_	0.7595	29.0843	23.5596	3	69.072
RFR_200H_	0.6784	32.9689	26.4953	2	64.948
RFR_200I_	0.6995	33.6597	27.3013	1	63.918

**Table 16:** Ranking values of RFR_100_.

Sensor	Ranking values in the following criterion					Weighted ranking values
	R^2^	RMSE	MAE	Variable utilization	Accuracy	
RFR_100A_	7	6	6	1	4	99
RFR_100B_	8	9	9	2	5	129
RFR_100C_	6	7	7	3	6	122
RFR_100D_	9	8	8	4	6	140
RFR_100E_	5	5	5	5	8	130
RFR_100F_	4	4	4	6	9	132
RFR_100G_	3	3	3	7	3	64
RFR_100H_	1	2	2	8	2	42
RFR_100I_	2	1	1	9	1	32

**Table 17:** Ranking values of RFR_200_.

Sensor	Ranking values in the following criterion					Weighted ranking values
	R^2^	RMSE	MAE	Variable utilization	Accuracy	
RFR_200A_	7	6	7	1	5	111
RFR_200B_	8	9	6	2	4	113
RFR_200C_	6	8	9	3	7	139
RFR_200D_	5	7	8	4	7	131
RFR_200E_	4	5	5	5	6	106
RFR_200F_	9	4	4	6	9	152
RFR_200G_	3	3	3	7	3	64
RFR_200H_	1	2	2	8	2	42
RFR_200I_	2	1	1	9	1	32

In random forests, each decision tree is generated independently by using bootstrap sampling and random feature selection to increase the diversity among the trees^23^. This is done to achieve better generalization ability and reduce variance.

However, if the dataset exhibits strong feature correlations or similar settings such as parameters and feature subsets are used during tree construction, the correlation among the decision trees in the random forest may increase. When there is high correlation among the decision trees, increasing the number of trees may not lead to significant improvement in performance.

This is because the strength of a random forest lies in having multiple independent decision trees that can form a more robust model. They reduce variance and mitigate overfitting risks by aggregating the individual predictions. But when the decision trees are highly correlated, they may make similar predictions and fail to provide additional diversity and information.

Therefore, when there is high correlation among the decision trees, increasing the number of trees may not bring noticeable improvements because of limited prediction variability among them. In such cases, alternative measures such as adjusting other hyperparameters, using feature selection methods, or trying different models may be necessary to enhance the model's performance.

### Comparison of different variable sets

**Figure 8:**
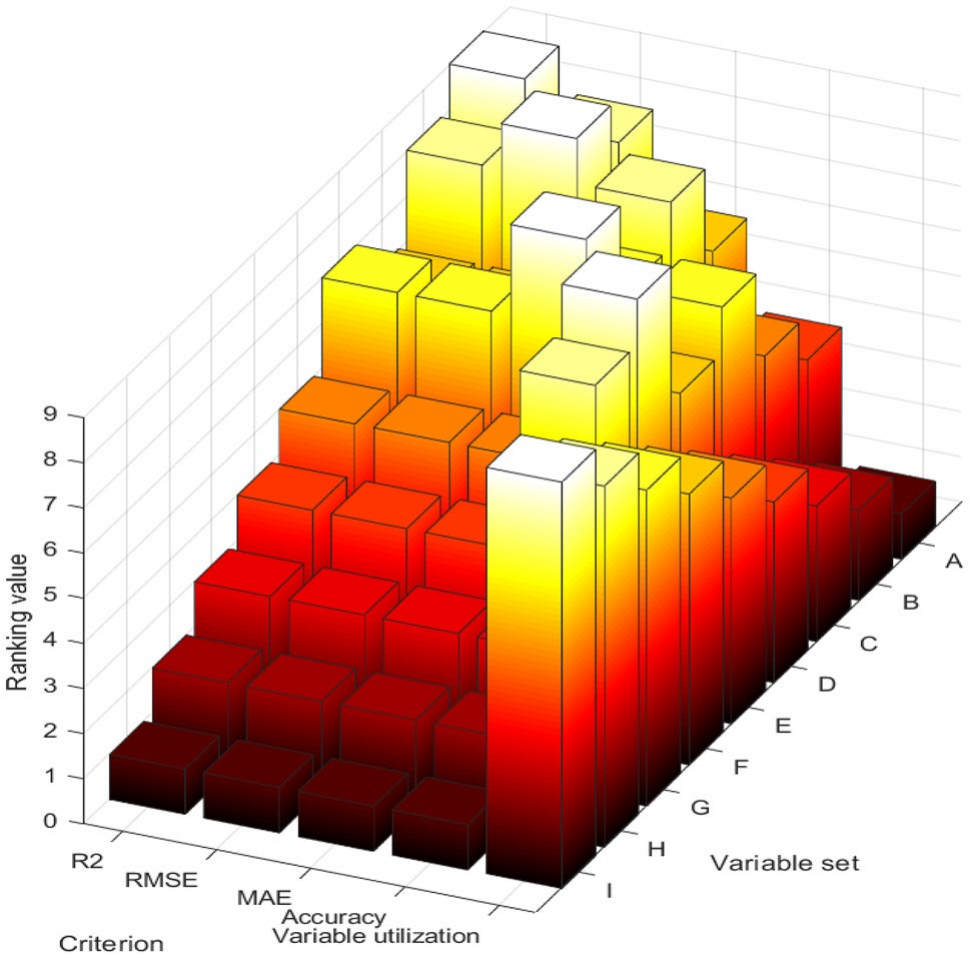
Ranking values of variable set A–I.

Different sets of variables lead to distinct prediction performances. A comparative evaluation was conducted on the performances of variable sets A to I, comprising nine different sets of variables, across various models. The results are obtained in Fig. 8 and Tables 18 and 19.

**Table 18:** Ranking values of variable set A-I.

Variable set	Ranking values in the following criterion(average value)					Weighted ranking values
	R^2^	RMSE	MAE	Variable utilization	Accuracy	
A	9	8	6	1	4	113
B	8	9	8	2	5	127
C	6	6	7	3	7	129
D	7	7	9	4	6	131
E	5	5	5	5	9	140
F	4	4	4	6	8	122
G	3	3	3	7	3	64
H	2	2	2	8	2	46
I	1	1	1	9	1	28

**Table 19:** Results of variable set A–I (average value).

Variable set	R^2^	RMSE	MAE	Variable utilization	Accuracy (%)
A	0.8322	26.38	20.92	9	71.85
B	0.8316	26.22	20.85	8	73.42
C	0.8246	26.61	20.89	7	74.04
D	0.8255	26.52	20.59	6	73.98
E	0.8199	26.71	21.01	5	76.23
F	0.8172	27.37	21.44	4	74.45
G	0.7856	27.80	22.27	3	67.43
H	0.7395	31.41	25.39	2	65.82
I	0.7193	32.56	26.34	1	59.24

The variable sets A to I contain 9–1 feature(s), with set A having nine input features and set I having only 1. These features were selected based on their Pearson correlation coefficients, for example, set B includes the top eight input features ranked by their correlation coefficients. In theory, having more input features in a variable set should lead to better prediction performance.However, indiscriminately increasing the number of input feature types can lead to overfitting and a decline in prediction performance. One possible reason for this is the presence of multicollinearity among the input features, where they exhibit strong linear relationships with each other.

After considering the weights, variable set E demonstrates exceptional performance across various software sensor models. Moreover, it attains the highest average prediction accuracy. Accurate prediction of ozone concentrations is vital for effective environmental monitoring. However, not all levels of ozone concentration are equally important. In areas with low concentrations considered safe, the emphasis on ozone decreases. Conversely, when ozone levels exceed local environmental standards, there is a heightened focus on ozone, demanding accurate predictions. This research distinguishes itself by prioritizing prediction accuracy during exceedance conditions, introducing innovative evaluation methods, and recognizing accuracy as a critical performance metric for virtual ozone sensors. The study aims to enhance our understanding of ozone exceedance and improve prediction capabilities for better environmental management.

We examine the distinction between variable sets E and A, B, C, D. While variable sets A, B, C, D incorporate an additional input feature, namely PM_10_, compared to variable set E, their overall performance falls short, particularly in terms of prediction accuracy. We postulate that this disparity can be attributed to the pronounced correlation between PM_10_ and PM_2.5_, indicating a significant presence of multicollinearity among the variables.

### Comparison of different models

**Figure 9:**
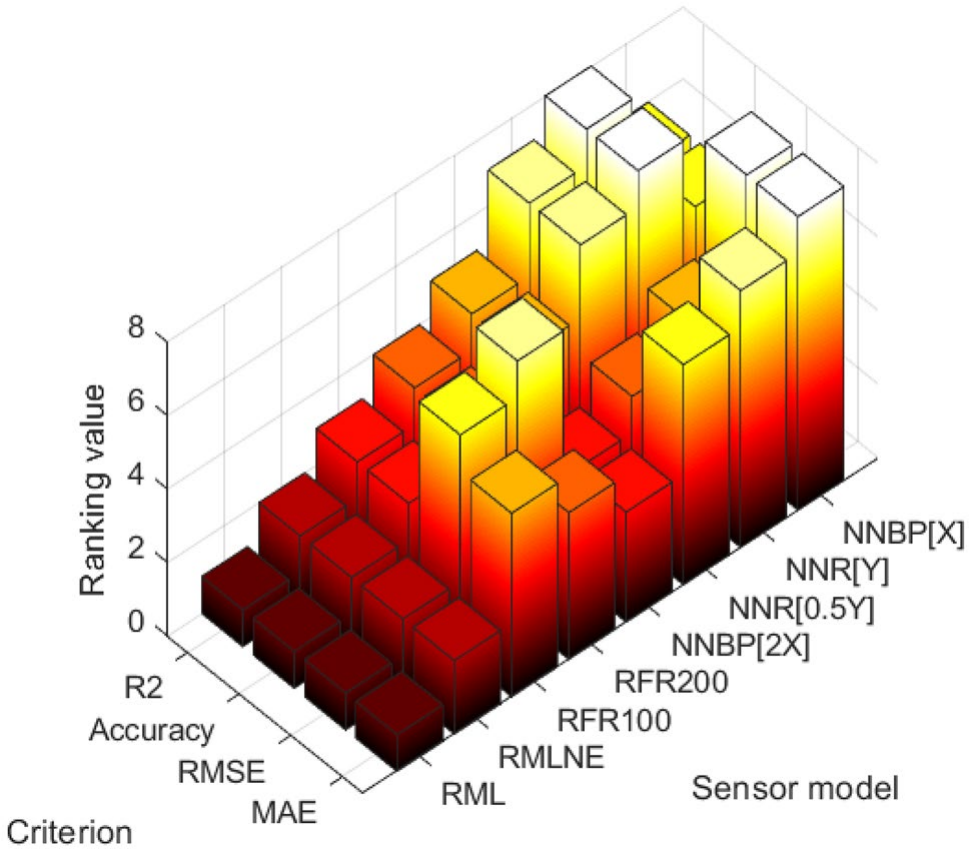
Ranking values of models.

Different sensor models lead to distinct prediction performances. A comparative evaluation was conducted on the performances of models, comprising eight different models, across various variable sets. The results obtained are as Fig. 9 and Tables 20 and 21.

**Table 20:** Ranking values of models.

Models	Ranking values in the following criterion(average value)					Weighted ranking values
	R^2^	RMSE	MAE	Variable utilization	Accuracy	
R_ML_	1	1	1	–	1	19
R_MLNE_	2	2	2	–	2	38
NN_BP[X]_	6	8	8	–	6	124
NN_BP[2X]_	5	3	3	–	5	85
NN_R[Y]_	8	5	7	–	8	141
NN_R[0.5Y]_	7	4	6	–	7	122
RFR_100_	3	6	5	–	3	70
RFR_200_	4	7	4	–	4	85

**Table 21:** Results of models (average value).

Models	R2	RMSE	MAE	Variable utilization	Accuracy (%)
R_ML_	0.7154	30.57	24.71	5	58.12
R_MLNE_	0.7310	29.72	24.01	5	59.16
NN_BP[X]_	0.8391	26.89	21.06	5	75.72
NN_BP[2X]_	0.8355	27.93	21.87	5	74.00
NN_R[Y]_	0.8559	27.21	21.26	5	75.95
NN_R[0.5Y]_	0.8493	27.27	21.43	5	75.94
RFR_100_	0.7812	27.09	21.54	5	73.43
RFR_200_	0.7886	26.98	21.64	5	73.43

Overall, NN model outperform RFR and LR models in several aspects. NN model has gained popularity due to their ability to learn complex patterns and relationships in data, making them highly effective for a wide range of tasks.

One key advantage of NN is their ability to capture nonlinear relationships between input features and target variables. Unlike LR models, which assumes a linear relationship, NN can model intricate nonlinear interactions, allowing them to capture more complex patterns in the data. This flexibility makes NN well-suited for tasks where the underlying relationships are nonlinear or involve interactions between multiple variables.

Furthermore, NN is highly flexible in terms of model architecture. They can be designed with multiple layers and a large number of neurons, allowing them to capture intricate relationships and handle high-dimensional data effectively. This adaptability enables NN to handle a wide range of data types, including text, images, and sequential data, making them suitable for various applications such as time series analysis.

However, it's important to note that NN also has certain limitations. It often requires a large amount of labeled training data to achieve optimal performance and can be computationally expensive to train and deploy. Additionally, NN is prone to overfitting if not properly regularized and may be challenging to interpret compared to simpler models like linear regression.

In summary, NN models offer significant advantages over RFR and LR models. Its ability to capture nonlinear relationships, automatically learn features, and adapt to diverse data types make them a powerful tool for solving complex machine learning problems. However, the choice of model should be based on the specific characteristics of the dataset, computational resources, interpretability requirements, and the trade-off between model complexity and performance.

In the realm of NN models, RNN surpasses BPNN neural network models. This is primarily due to the distinct network architectures they employ. Specifically, RNN exhibit a remarkable advantage in handling time series data, which is evident in this particular scenario.

RNN excel at capturing temporal dependencies and patterns by incorporating memory units and recurrent connections. These architectural elements allow the model to retain and leverage information from past observations, thereby enhancing its ability to predict future outcomes. On the other hand, BPNN may encounter challenges such as information loss and gradient vanishing when confronted with time series data, as they independently process inputs at each time step.

Moreover, RNN possess the capability to handle variable-length sequence data by iteratively updating hidden states. This flexibility proves particularly advantageous when dealing with time series data of varying lengths, as RNN can adapt to the unique characteristics and patterns exhibited by different sequences.

Overall, by judiciously selecting NN models, particularly RNN, one can attain more accurate and reliable predictions. This approach offers an effective means of improving prediction performance and enhancing decision-making accuracy in similar cases.

### Comparison of all sensors

Here is the ranking of the 72 sensors based on their comprehensive predictive performance, taking into account the weights and comparisons across various criterion (Table 22).

**Table 22:** Ranking values of all sensors.

Variable set	Ranking values in the following criterion					Weighted ranking values
	R^2^	RMSE	MAE	Variable utilization	Accuracy	
NN_R[Y]C_	69	62	69	7	66	1267
NN_BP[X]E_	58	71	71	5	66	1252
NN_R[Y]E_	67	46	60	5	70	1231
NN_BP[2X]E_	53	52	50	5	72	1193
NN_R[0.5Y]B_	68	59	55	8	62	1187
NN_R[Y]D_	70	51	48	6	62	1155
NN_R[Y]B_	72	70	72	8	49	1140
NN_BP[X]C_	60	38	37	7	70	1135
NN_R[0.5Y]E_	64	37	35	5	69	1132
NN_BP[X]D_	54	64	70	6	57	1124
NN_R[0.5Y]C_	65	41	44	7	62	1098
RFR_100F_	32	57	56	4	66	1075
RFR_200F_	49	55	51	4	57	1037
NN_BP[2X]D_	62	33	53	6	57	1029
NN_R[0.5Y]F_	56	44	49	4	57	1028
NN_R[Y]A_	71	48	47	9	49	1021
NN_BP[X]B_	61	36	40	8	57	1010
RFR_200C_	41	69	67	7	49	1002
NN_R[Y]F_	50	35	34	4	62	997
NN_R[0.5Y]D_	52	54	59	6	49	984
RFR_200D_	38	67	64	6	49	977
NN_BP[2X]B_	66	42	42	8	49	972
NN_BP[2X]A_	63	56	54	9	42	957
RFR_100E_	33	60	58	5	49	923
RFR_100D_	42	66	65	6	42	922
RFR_200B_	45	72	62	8	39	918
NN_R[0.5Y]A_	55	40	39	9	49	917
NN_BP[X]F_	57	50	52	4	42	906
NN_BP[X]A_	59	49	46	9	42	904
RFR_100B_	39	68	68	8	40	904
RFR_200E_	35	58	57	5	47	903
RFR_100C_	36	65	66	7	42	898
RFR_200A_	43	63	63	9	40	896
RFR_100A_	40	61	61	9	38	854
NN_BP[2X]F_	51	32	33	4	47	840
NN_R[Y]G_	48	39	36	3	36	744
NN_BP[X]G_	37	47	43	3	36	738
NN_R[0.5Y]G_	46	45	41	3	30	704
NN_BP[2X]C_	47	43	38	7	30	700
NN_BP[2X]G_	34	53	45	3	30	688
RFR_100G_	26	31	31	3	28	542
RFR_200G_	27	30	30	3	28	541
NN_R[0.5Y]H_	44	16	16	2	27	528
NN_BP[X]H_	29	19	22	2	30	519
NN_R[Y]H_	31	15	17	2	30	505
NN_BP[2X]H_	30	9	9	2	30	467
R_MLNEC_	22	28	28	7	17	405
NN_R[0.5Y]I_	21	8	10	1	25	379
NN_BP[X]I_	25	11	11	1	21	366
R_MLNEG_	12	20	24	3	19	349
R_MLNEA_	28	34	32	9	5	337
R_MLNEB_	20	27	25	8	11	329
R_MLD_	18	26	26	6	11	318
R_MLC_	17	25	27	7	11	314
R_MLNED_	23	29	29	6	6	303
RFR_100H_	1	6	7	2	26	298
R_MLNEH_	9	14	13	2	19	296
R_MLNEF_	16	24	21	4	11	292
R_MLB_	14	22	23	8	11	286
R_MLE_	8	13	12	5	18	280
RFR_200H_	2	5	5	2	24	275
R_MLA_	15	23	18	9	9	264
R_MLNEE_	13	21	20	5	10	260
RFR_200I_	5	3	3	1	21	246
RFR_100I_	6	2	2	1	21	245
R_MLF_	7	12	14	4	11	206
NN_R[Y]I_	24	4	4	1	7	187
R_MLG_	10	17	19	3	4	172
NN_BP[2X]I_	19	1	1	1	7	152
R_MLNEI_	11	18	15	1	1	139
R_MLI_	4	10	8	1	2	83
R_MLH_	3	7	6	2	3	77

**Figure 10:**
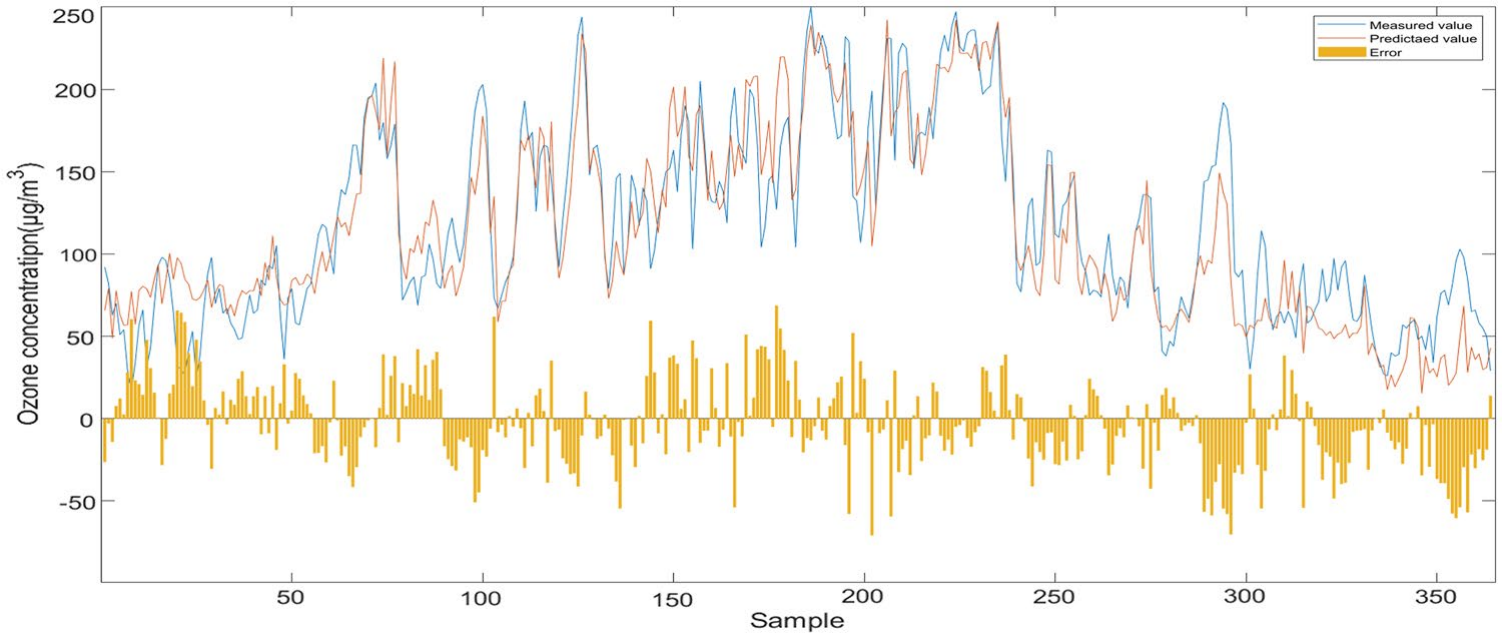
Prediction result of sensor NN_R[Y]C_.

Among the 72 sensors evaluated, NN_R[Y]C_ exhibits exceptional performance, boasting an impressive R^2^ of 0.8902, a low RMSE of 24.91, and an equally impressive MAE of 19.16. Notably, this sensor achieves an outstanding prediction accuracy of 81.44%, further enhancing its credibility and reliability. These remarkable results position NN_R[Y]C_ as a top-performing sensor, making it a compelling choice for various technological applications.Here are the prediction result of sensor NN_R[Y]C_ on the test set, demonstrating its effectiveness in accurately forecasting outcome(Fig. 10).

## Conclusion

### Summary of the study

In the proposed methodology, we conducted a comprehensive analysis of soft sensor modeling techniques for air ozone prediction. We compared the performance of three different modeling techniques: LR (linear regression), NN (neural networks), and RFR (random forest regression). Additionally, we evaluated the impact of different variable sets on prediction performance.

### Discussion of the most effective modeling technique

Based on our findings, we conclude that neural network models, particularly the RNN (recurrent neural network) variant, outperformed the other modeling techniques in terms of prediction accuracy. RNN demonstrated superior capabilities in capturing temporal dependencies and patterns in time series data, making them highly effective for air ozone prediction. The flexibility of RNN in handling variable-length sequences further enhances their performance in modeling dynamic environmental processes.

### Future directions for research in soft sensor modeling for air ozone prediction

While the proposed methodology provides valuable insights into soft sensor modeling for air ozone prediction, there are several areas that warrant further investigation.

1. Enhanced model interpretability: NN, although highly effective, is often considered black-box models, making it challenging to interpret their predictions. Future research should focus on developing techniques to improve the interpretability of NN models, enabling a better understanding of the underlying relationships between input variables and ozone concentrations.
2. Integration of domain knowledge: Incorporating domain knowledge and expert insights into the modeling process can enhance the accuracy and reliability of soft sensor models. Future research should explore methods for effectively integrating domain knowledge into the modeling framework, such as utilizing physical laws and environmental factors that influence ozone concentrations.
3. Ensemble modeling approaches: Ensemble modeling techniques, such as combining multiple models or incorporating expert knowledge, have shown promise in improving prediction accuracy. Future research should investigate the potential benefits of ensemble modeling for air ozone prediction, exploring ways to leverage the strengths of different modeling techniques and variable sets.
4. Real-time monitoring and feedback: Developing soft sensor models that can provide real-time monitoring and feedback on ozone concentrations is essential for effective environmental management. Future research should focus on developing online learning algorithms that can continuously update the soft sensor models and adapt to changing environmental conditions in real-time.
5. Generalizability and transferability: It is important to assess the generalizability and transferability of soft sensor models across different geographical locations and time periods. Future research should explore methods for evaluating the robustness and transferability of soft sensor models, considering variations in environmental conditions, sensor configurations, and data availability.

In conclusion, the proposed methodology highlights the superiority of neural network models, particularly recurrent neural networks, for soft sensor modeling in air ozone prediction. The findings provide valuable insights for researchers and practitioners in the field of environmental monitoring and management. Future research should focus on enhancing model interpretability, integrating domain knowledge, exploring ensemble modeling approaches, enabling real-time monitoring and assessing model generalizability as well as transferability. These advancements will contribute to the development of more accurate and reliable soft sensor models for air ozone prediction, ultimately supporting effective environmental management strategies.

## Data Availability

The datasets used and/or analysed during the current study available from the corresponding author on reasonable request.
